# Isoallolithocholic acid attenuates acute liver allograft rejection by promoting Treg differentiation through the NR4A1/FOXP3 signaling axis

**DOI:** 10.3389/fphar.2026.1953528

**Published:** 2026-10-27

**Authors:** Ruiheng Luo, Hanfei Huang, Qianfei Zuo, Zhong Zeng, Tengqian Tang

**Affiliations:** 1 Department of Hepatobiliary Surgery, First Affiliated Hospital, Army Medical University, Chongqing, China; 2 The First Affiliated Hospital of Kunming Medical University, Kunming, China; 3 Yunnan Key Laboratory of Organ Transplantation, Kunming, China; 4 Institute of Xenotransplantation, Kunming Medical University, Kunming, China; 5 Department of Microbiology and Biochemical Pharmacy, College of Pharmacy, Army Medical University, Chongqing, China

**Keywords:** acute allograft rejection, immune tolerance, isoallolithocholic acid, NR4A1, orthotopic liver transplantation, regulatory T cells

## Abstract

**Background:**

Acute allograft rejection driven by disordered CD4^+^ T cell alloimmunity severely limits long-term graft survival following orthotopic liver transplantation (OLT). Conventional broad-spectrum immunosuppressants cause severe systemic side effects, while therapeutic approaches relying on metabolites to induce transplant tolerance remain insufficiently explored. Isoallolithocholic acid (isoallo-LCA), a secondary bile acid produced by gut microbiota, maintains Treg homeostasis, yet its biological function and molecular mechanisms in regulating hepatic alloimmune rejection have not been clarified.

**Objective:**

To systematically explore the therapeutic efficacy of isoallo-LCA against acute liver allograft rejection and uncover its underlying molecular regulatory mechanism.

**Methods:**

An MHC-mismatched murine orthotopic liver transplantation acute rejection model was established. Recipient survival, liver pathological injury, BANFF rejection scores and serum hepatic injury indicators (ALT, AST, TBIL) were detected to evaluate the protective effect of isoallo-LCA. Flow cytometry was applied to analyze intrahepatic Th1 and Treg proportions. Transcriptomic sequencing combined with *in vivo* and *in vitro* western blot assays was used to screen and verify key signaling molecules, and an NR4A1 inhibitor was administered to conduct rescue validation experiments.

**Results:**

Isoallo-LCA intervention significantly prolonged recipient survival, alleviated intrahepatic inflammatory damage, lowered BANFF rejection grades and decreased serum ALT, AST and TBIL levels. Isoallo-LCA inhibited the differentiation of pro-inflammatory T-bet^+^ Th1 cells and promoted the expansion of FOXP3^+^ Tregs, restoring the balance of intrahepatic CD4^+^ T cell subsets. NR4A1 was identified as the core downstream effector of isoallo-LCA. Blockade of NR4A1 pharmacologically nearly eliminated the protective phenotypes mediated by isoallo-LCA, confirming the essential role of the NR4A1/FOXP3 signaling pathway.

**Conclusion:**

Isoallo-LCA relieves acute liver allograft rejection by activating the NR4A1/FOXP3 signaling axis to remodel intrahepatic Th1/Treg immune balance. These findings provide preclinical evidence supporting gut microbial bile acid metabolites as potential low-toxic adjuvant candidates for inducing transplant immune tolerance, although clinical translation requires further validation in human studies.

## Introduction

Liver transplantation is currently the most effective therapeutic strategy for end-stage liver diseases, including liver failure and advanced liver cirrhosis (EASL clinical practice guidelines on liver transplantation, 2024; Zhou et al., 2024). Nevertheless, acute allograft rejection is a major postoperative complication that severely compromises graft function and long-term survival of recipients (Zhou et al., 2026; Zhao et al., 2022). Acute hepatic allograft rejection is predominantly driven by dysregulated alloimmune responses mediated by CD4^+^ T cell subsets (Ronca et al., 2020; Li et al., 2026). The imbalance between pro-inflammatory effector T cells and immunosuppressive regulatory T cells (Tregs) triggers persistent inflammatory infiltration, hepatocellular injury, and irreversible graft tissue damage (Ronca et al., 2020; Li et al., 2025). Although clinical broad-spectrum immunosuppressants can partially alleviate rejection, their non-specific immune suppression elevates the risks of postoperative infection, metabolic disorders, and other adverse events (Mehtani and Saigal, 2023; Rabiee et al., 2023). Therefore, novel targeted immunomodulatory molecules are urgently required to optimize immune tolerance after liver transplantation.

Isoallolithocholic acid (isoallo-LCA), a centenarian-enriched gut microbial secondary bile acid, displays pleiotropic bioactivities. It selectively kills multidrug-resistant Gram-positive bacteria to preserve intestinal immune homeostasis as a key immunomodulatory metabolite. Notably, isoallo-LCA governs immune cell differentiation, remodels intestinal immunity and suppresses systemic inflammation, with great potential to treat inflammatory disorders and induce immune tolerance. (Sato et al., 2021).

Despite the well-characterized immunomodulatory and tolerance-regulating properties of isoallo-LCA, its modulatory roles in solid organ alloimmunity, particularly during liver transplant rejection, remain largely uncharacterized. No prior studies have addressed three key questions: whether isoallo-LCA can interfere with alloimmune responses, alleviate acute rejection, and extend graft survival after liver transplantation; whether isoallo-LCA remodels the balance of intrahepatic immune cell subsets to establish a tolerogenic microenvironment; and the underlying molecular mechanisms mediating its immune regulatory effects. Given the essential regulatory roles of bile acid metabolites in immune homeostasis and the unique tolerance-inducing characteristics of isoallo-LCA, we hypothesized that isoallo-LCA may serve as a novel protective metabolite against liver transplant rejection. In the present study, we systematically investigated the therapeutic effects of isoallo-LCA on acute hepatic allograft rejection using a murine orthotopic liver transplantation (OLT) model (Yokota et al., 2025; Yokota et al., 2016). We clarified its regulatory function on intrahepatic CD4^+^ T cell subset balance and further uncovered the core NR4A1/FOXP3 signaling axis responsible for its immune tolerance induction. This study aims to provide novel theoretical evidence and potential therapeutic targets for the clinical prevention and treatment of acute liver allograft rejection.

Notably, conventional immunosuppressants, including calcineurin inhibitors (e.g., tacrolimus and cyclosporine), antimetabolites (e.g., mycophenolate mofetil), and mTOR inhibitors (e.g., sirolimus), exert their anti-rejection effects primarily through global suppression of T cell activation and proliferation (Mehtani and Saigal, 2023; Rabiee et al., 2023). While effective in controlling acute rejection, this non-specific immune suppression compromises systemic immune surveillance, elevating the risks of opportunistic infections, malignancy, and metabolic complications (Lan et al., 2025; Kensiski et al., 2025). In contrast, bile acid metabolites have emerged as a class of endogenous immunomodulators that selectively regulate immune cell differentiation and function without causing generalized immunosuppression (Sato et al., 2021). Among these, isoallo-LCA is distinguished by its ability to promote regulatory T cell differentiation while suppressing pro-inflammatory effector responses, suggesting a homeostatic rather than ablative mechanism of immune modulation. This unique pharmacological profile positions isoallo-LCA as a promising candidate for achieving the delicate balance between rejection control and immune competence that is the ultimate goal of transplant medicine.

## Materials and methods

### Animal models and ethical approval

Inbred male C57BL/6 and C3H mice aged 8–10 weeks were purchased from Beijing Vital River Laboratory Animal Technology Co., Ltd. and used to establish a murine orthotopic liver transplantation acute allograft rejection model. C57BL/6 mice served as liver donors, and C3H mice were used as transplant recipients. All experimental animals were housed under specific pathogen-free (SPF) conditions at a constant temperature of 22 °C–24 °C and relative humidity of 50%–60%, with a 12 h light/dark cycle. Mice had free access to standard laboratory chow and sterile drinking water. All animal experimental procedures strictly complied with the National Institutes of Health (NIH) guidelines for the care and use of laboratory animals. This study was approved by the Laboratory Animal Welfare and Ethics Review Committee of the First Affiliated Hospital of Kunming Medical University (No. 2026DF037).

### Orthotopic liver transplantation and isoallo-LCA intervention

The murine OLT model was established using the classic microsurgical cuff anastomosis technique as previously described. Briefly, donor liver grafts were harvested and perfused with cold sterile organ preservation solution, followed by orthotopic implantation into anesthetized recipient mice. After surgery, recipient mice were randomly assigned to three experimental groups: vehicle Control group, isoallo-LCA monotherapy group, and isoallo-LCA combined with NR4A1 inhibitor treatment group. For drug intervention, isoallo-LCA (purity ≥ 98%, as determined by HPLC; MedChemExpress, Cat# HY-N14535) was dissolved in sterile phosphate-buffered saline (PBS) at a working concentration of 2 mg/mL and intraperitoneally injected at a dose of 10 mg/kg body weight once daily between 09:00 and 10:00 a.m. from postoperative day (POD) 0 to POD14. Mice in the Control group received an equal volume of sterile PBS via intraperitoneal injection. Mice in the combined treatment group were administered the NR4A1 inhibitor 1,10-phenanthroline monohydrate monohydrate (Sigma-Aldrich, Cat# 131377) at a dose of 5 mg/kg body weight through daily intraperitoneal injection throughout the 14-day intervention period. Recipient survival status was continuously monitored and recorded for Kaplan–Meier survival analysis. Liver graft tissues and peripheral blood samples were collected on POD3, POD7, and POD14 for subsequent histological, biochemical, cellular, and molecular assays.

### Serum biochemical analysis

Peripheral blood samples were collected from the orbital venous plexus of recipient mice at designated postoperative time points. Blood samples were kept at room temperature for natural coagulation and centrifuged at 3,000 rpm for 15 min to isolate serum. Serum alanine aminotransferase (ALT), aspartate aminotransferase (AST), and total bilirubin (TBIL) levels were detected using an automatic biochemical analyzer following the manufacturer’s standard protocols to evaluate the severity of hepatocellular injury and acute allograft rejection.

### Histopathological examination and BANFF rejection scoring

Harvested liver allograft tissues were fixed in 4% paraformaldehyde solution (Solarbio, Cat# P1110), subjected to routine dehydration, paraffin embedding, and serially sectioned at a thickness of 5 μm. Standard hematoxylin and eosin (H&E) staining kits (Beyotime, Cat# C0105) were applied to visualize histopathological lesions, including inflammatory cell infiltration, interstitial edema, and hepatic parenchymal necrosis. Histopathological images were captured under an optical microscope. Two experienced pathologists independently evaluated and scored the severity of acute liver allograft rejection in a blinded manner according to the BANFF acute liver rejection grading criteria. Quantitative rejection scores were calculated and statistically analyzed to assess the degree of allograft tissue injury.

### Flow cytometry of graft-infiltrating CD4^+^ T Cell subsets

Liver graft tissues were fully homogenized and filtered through 70 μm cell strainers (Corning, Cat# 352350) to prepare single-cell suspensions. Graft-infiltrating lymphocytes were isolated via density gradient centrifugation using mouse lymphocyte separation medium (Dakewe, Cat# DKW33-R0100). For CD4^+^ T cell subset detection, cell surface staining was performed at 4 °C for 30 min with anti-mouse CD4-FITC (BD Biosciences, Cat# 553047, 1:200), followed by fixation and permeabilization with Fix/Perm buffer (BD Biosciences, Cat# 554722) and intracellular transcription factor staining at 4 °C for 40 min using anti-mouse T-bet-PE (eBioscience, Cat# 12-5825-82, 1:150) and anti-mouse FOXP3-APC (eBioscience, Cat# 17-5773-82, 1:150). Lymphocytes were first identified by forward/side scatter properties, single cells were gated using FSC-H vs. FSC-A, and CD4^+^ T cells were subsequently identified. Th1 cells were defined as T-bet+CD4^+^ and Tregs as FOXP3+CD4^+^. A minimum of 10,000 CD4^+^ events were acquired per sample. T-bet^+^CD4^+^ Th1 cells and FOXP3^+^CD4^+^ regulatory T cells (Tregs) were quantified on a BD LSRFortessa™ cell analyzer, and subset proportions were analyzed via FlowJo™ software v10.

### Western blot

Total proteins from liver allograft tissues and primary CD4^+^ T cells were extracted with RIPA lysis buffer supplemented with 1 × protease inhibitor cocktail (Roche, Cat# 4693132001). Protein concentration was determined using a BCA kit (Thermo Fisher, Cat# 23225). Equal 30 μg protein samples were separated by 10% SDS-PAGE and transferred to PVDF membranes (Millipore, Cat# IPVH00010). After 1 h blocking with 5% skimmed milk, membranes were incubated overnight at 4 °C with primary antibodies: anti-NR4A1 (Abcam, Cat# ab109180, 1:1000), anti-FOXP3 (CST, Cat# 12653S, 1:1000), anti-GAPDH (Proteintech, Cat# 10494-1-AP, 1:2000). Blots were incubated with HRP-conjugated goat anti-rabbit IgG (Jackson, Cat# 111–035–003, 1:5,000) for 1 h at room temperature. Protein bands were visualized with ECL reagent (Millipore, Cat# WBKLS0100); GAPDH served as internal reference, and relative expression was quantified with ImageJ.

### Isolation and *in vitro* culture of primary CD4^+^ T Cells

Primary CD4^+^ T lymphocytes were isolated from mouse spleens using magnetic bead sorting kits (Miltenyi Biotec, Cat# 130–104–454). Purified cells were cultured in RPMI-1640 complete medium at 37 °C with 5% CO_2_ and divided into three groups: vehicle PBS control, 5 ng/mL TGF-β1 (PeproTech, Cat# 100–21) single treatment, and combined treatment with 5 ng/mL TGF-β1 plus 10 μM isoallo-LCA. After 72 h incubation, cells were collected for western blot analysis of NR4A1 and FOXP3 expression.

### Transcriptomic sequencing and KEGG pathway enrichment analysis

Total RNA was extracted from liver graft tissues of vehicle Control and isoallo-LCA-treated mice using the TRIzol Reagent kit (Invitrogen, Cat# 15596026). RNA integrity was assessed using an Agilent 2,100 Bioanalyzer, and RNA quantification was performed using a Qubit 4.0 Fluorometer. RNA libraries were constructed using the NEBNext Ultra II RNA Library Prep Kit for Illumina (New England Biolabs, Cat# E7770). Sequencing was performed on the Illumina NovaSeq 6,000 platform (PE150). Raw reads were quality-filtered using fastp (v0.23.4) and aligned to the GRCm39 mouse reference genome using STAR (v2.7.11b). Gene-level read counts were quantified using featureCounts (v2.0.5). DEGs were identified using DESeq2 (v1.40.2) in R (v4.3.2); genes with adjusted P value (Benjamini-Hochberg FDR) < 0.05 and |log2 (fold change)| > 1 were considered significantly differentially expressed. KEGG pathway enrichment analysis was performed using clusterProfiler (v4.8.3) in R, with adjusted P value < 0.05 as the significance threshold.

### Statistical analysis

All experimental data in this study are presented as the mean ± standard error of the mean (SEM). Kaplan–Meier survival curves were plotted, and the log-rank test was applied for inter-group survival difference analysis. Unpaired Student’s t-test was used for statistical comparisons between two groups, and one-way analysis of variance (ANOVA) followed by Tukey’s *post hoc* test was adopted for multiple-group comparisons. All statistical analyses were performed using GraphPad Prism 8.0 (GraphPad Software, San Diego, CA, United States) and SPSS version 26.0 (IBM Corp., Armonk, NY, United States). *P* value < 0.05 was considered statistically significant.

## Results

### Isoallo-LCA ameliorates acute allograft injury and prolongs liver transplant survival in mice

To define the biological role of isoallo-LCA in hepatic alloimmunity, recipient mice undergoing orthotopic liver transplantation received continuous isoallo-LCA or vehicle control treatment postoperatively. Kaplan–Meier survival analysis showed that isoallo-LCA prolonged liver allograft survival relative to vehicle-treated animals (log-rank test, P = 0.012) (Figure 1A).

**FIGURE 1:**
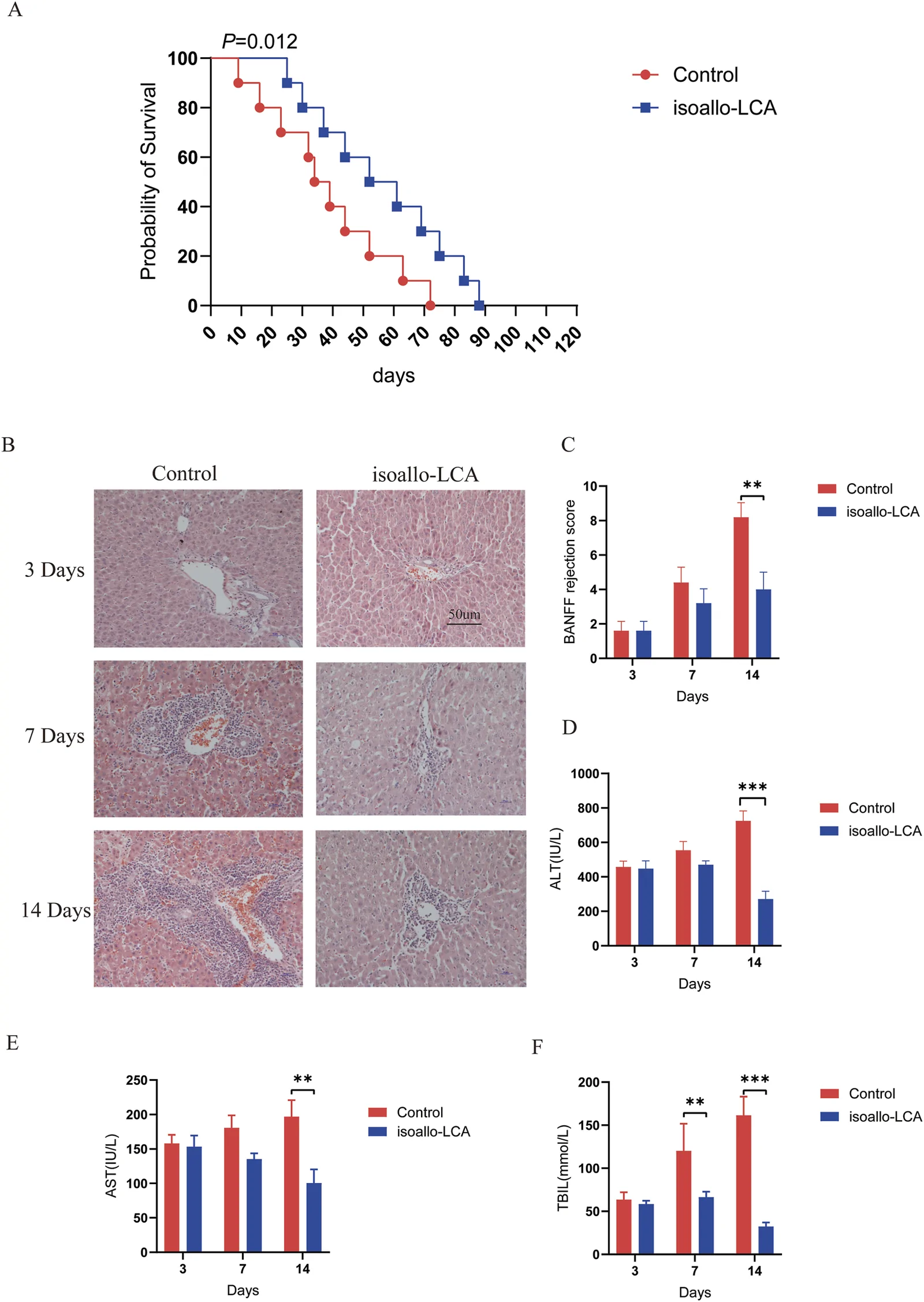
Isoallo-LCA mitigates acute rejection and improves survival in mouse orthotopic liver allografts. **(A)** Kaplan–Meier survival curves for vehicle Control and isoallo-LCA-treated recipient mice (n = 10 mice per group). Isoallo-LCA treatment significantly prolonged liver allograft survival (*P* = 0.012, log-rank test). **(B)** Representative H&E-stained histological images of liver allografts harvested on POD3, POD7 and POD14 post-transplantation. Isoallo-LCA reduced inflammatory cell infiltration and hepatic parenchymal tissue damage 50 μm. **(C)** Quantitative statistical analysis of BANFF acute rejection scores (n = 5 mice per group). Rejection scores were markedly lower in the isoallo-LCA group at POD14 (**P < 0.01 vs. Control). **(D–F)** Serum levels of hepatocellular injury markers ALT, AST and TBIL detected at POD3, POD7 and POD14. Isoallo-LCA markedly reduced the elevation of liver injury biomarkers induced by acute rejection (**P < 0.01, ***P < 0.001 vs. Control).

Histopathological assessment of graft tissues collected at POD3, POD7 and POD14 showed severe inflammatory infiltration and parenchymal necrosis in the control group, whereas isoallo-LCA treatment relieved tissue lesions at all examined time points (Figure 1B). In line with these morphological changes, quantitative BANFF scoring indicated milder rejection injury in the isoallo-LCA group at POD14 (P < 0.01) (Figure 1C).

Serum liver function markers further supported the hepatoprotective potential of isoallo-LCA. The high levels of ALT, AST and TBIL reflecting severe hepatocyte damage in control mice declined notably after isoallo-LCA administration (P < 0.01, P < 0.001) (Figures 1D-F). Collectively, these results suggest that isoallo-LCA may relieve acute liver allograft injury and help improve graft prognosis after transplantation.

### Isoallo-LCA regulates the balance of Th1/Treg cells

To investigate whether isoallo-LCA remodels the balance of CD4^+^ T cell subsets after liver transplantation, flow cytometry was performed to quantify the proportions of T-bet^+^CD4^+^ Th1 cells and FOXP3^+^CD4^+^ Tregs in liver grafts harvested from Control and isoallo-LCA-treated mice (Figure 2).

**FIGURE 2:**
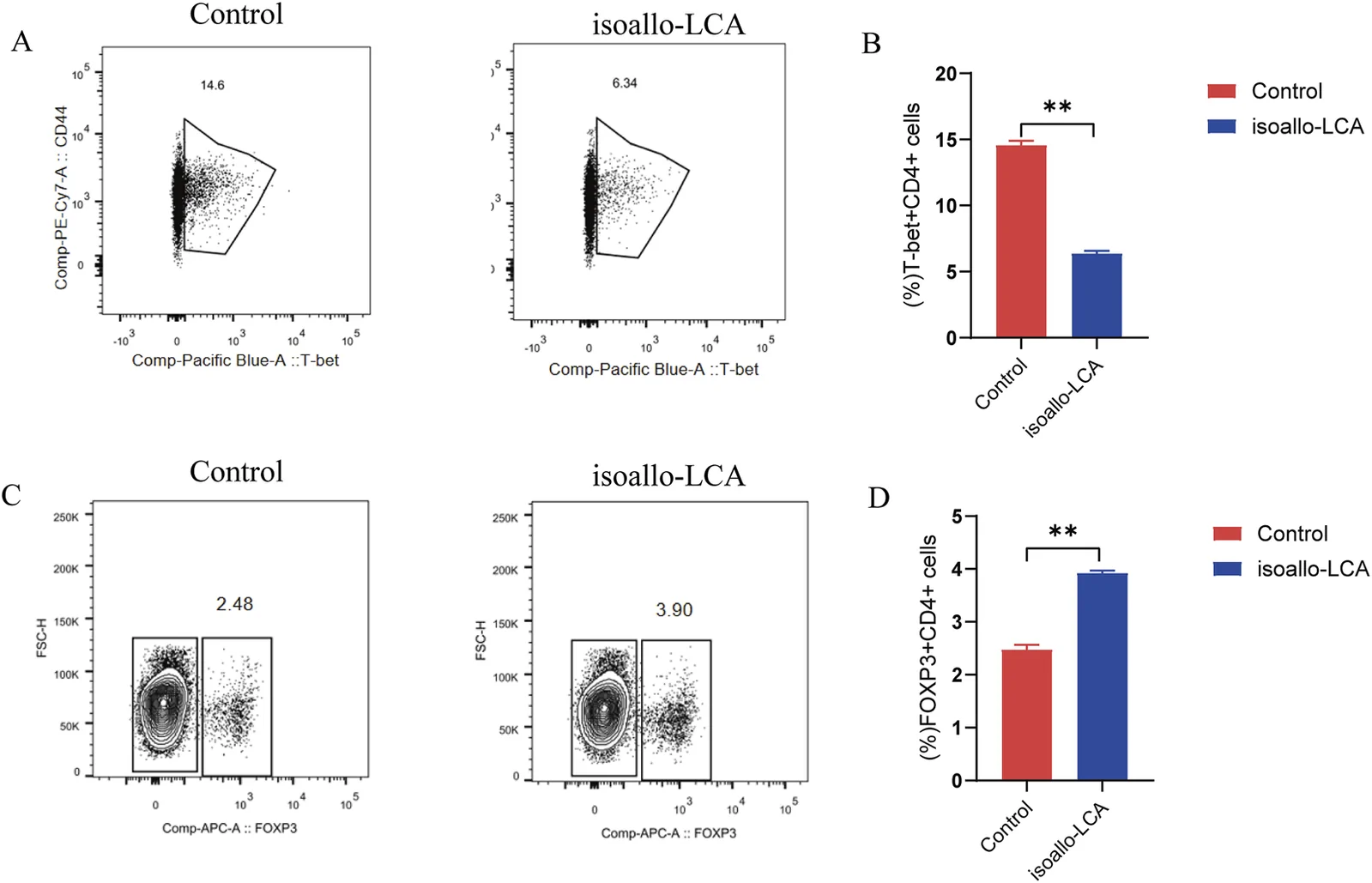
Isoallo-LCA suppresses Th1 cell differentiation and promotes Treg cell generation in a mouse liver allograft model. **(A)** Representative flow cytometry dot plots showing the frequency of T-bet^+^CD4^+^ Th1 cells in the Control group and isoallo-LCA treatment group. Numerical values within plots indicate the percentage of T-bet^+^CD4^+^ cells. **(B)** Quantitative statistical analysis of the proportion of T-bet^+^CD4^+^ Th1 cells from flow cytometry data (n ≥ 3 biological replicates per group). Compared with the Control group, isoallo-LCA significantly reduced the percentage of intrahepatic Th1 cells. **(C)** Representative flow cytometry contour plots displaying the percentage of FOXP3^+^CD4^+^ regulatory T cells (Tregs) in Control and isoallo-LCA groups. Numerical values within plots represent the proportion of FOXP3^+^CD4^+^ cells. **(D)** Statistical quantification of FOXP3^+^CD4^+^ Treg cell percentages (n ≥ 3 biological replicates per group). Isoallo-LCA treatment markedly elevated intrahepatic Treg frequency relative to the Control group. All data are presented as mean ± standard error of the mean (SEM). **P < 0.01 versus Control group.

The frequency of intrahepatic T-bet^+^CD4^+^ Th1 cells was 14.57% in Control mice, which declined markedly to 6.38% after isoallo-LCA administration (Figures 2A,B, *P* < 0.01), indicating that isoallo-LCA effectively suppresses Th1 differentiation within liver allografts.

We next assessed the frequency of FOXP3^+^ regulatory T cells (Tregs). As shown in the contour plots (Figure 2C), FOXP3^+^CD4^+^ Tregs accounted for 2.48% of intrahepatic CD4^+^ T cells in Control mice, and isoallo-LCA treatment raised this value to 3.92% (Figure 2D, *P* < 0.01), suggesting that isoallo-LCA may facilitate Treg expansion within liver allografts.

Taken together, these flow cytometric data indicate that isoallo-LCA suppresses the differentiation of pro-inflammatory Th1 cells and simultaneously promotes the generation of immunosuppressive Tregs within liver allografts, thereby shifting the intrahepatic CD4^+^ T cell balance toward an immune-tolerant phenotype.

### Isoallo-LCA induces NR4A1 activation to sustain FOXP3 expression and maintain CD4^+^ T-cell homeostasis

NR4A1 (Nur77) is an orphan nuclear receptor belonging to the NR4A subfamily that functions as a critical transcription factor in T cell development, activation, and differentiation. Previous studies have demonstrated that NR4A1 promotes FOXP3 expression and regulatory T cell differentiation by binding directly to the FOXP3 promoter region. To delineate the mechanism by which isoallo-LCA modulates Treg homeostasis, we performed KEGG pathway enrichment analysis based on transcriptomic alterations induced by isoallo-LCA treatment (Figure 3A). The NR4A1-associated signaling cascade showed the most significant enrichment, suggesting NR4A1 may act as a key downstream effector of isoallo-LCA. Western blot assays of liver allograft tissues further revealed that isoallo-LCA steadily elevated NR4A1 and FOXP3 protein expression across POD3 to POD14 (Figure 3B).

**FIGURE 3:**
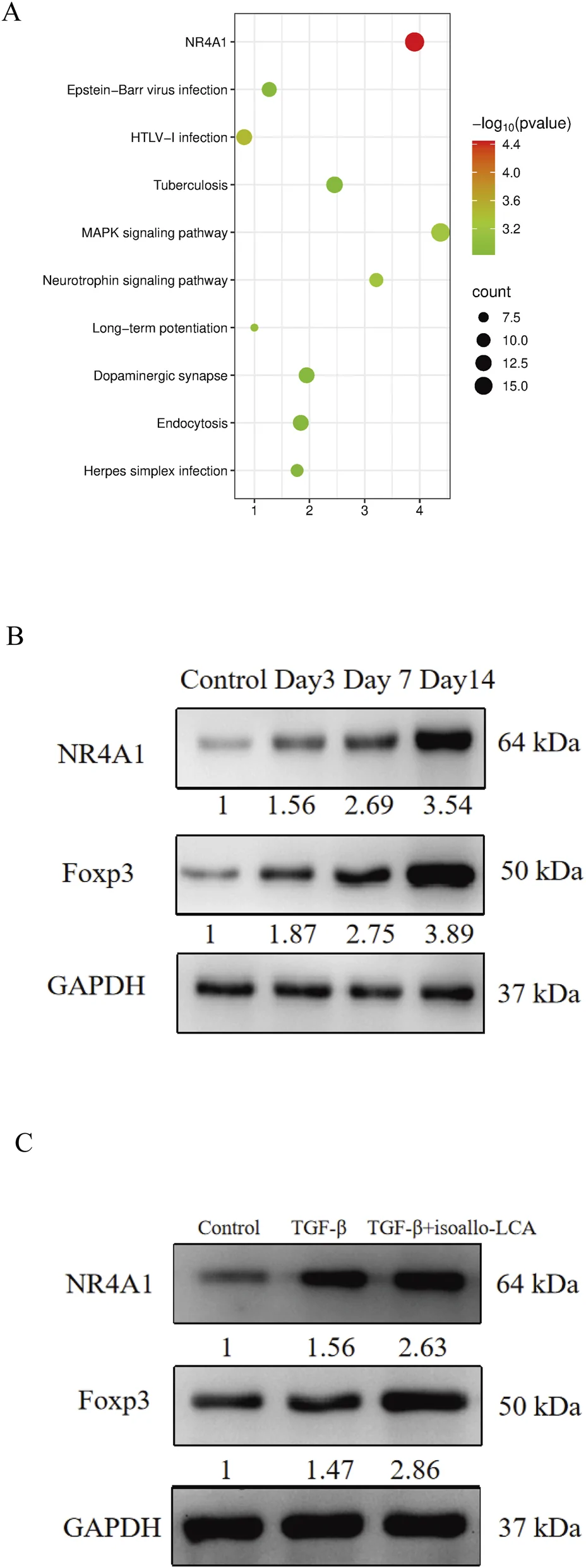
Isoallo-LCA activates NR4A1 to upregulate FOXP3 and maintain CD4^+^ T cell homeostasis. **(A)** Bubble plot of KEGG pathway enrichment for isoallo-LCA-induced differentially expressed genes. X-axis: enrichment score; Y-axis: annotated signaling pathways; dot size: number of enriched genes; dot color: −log_10_(*P* value). **(B)** Western blot analysis of NR4A1 (64 kDa) and FOXP3 (50 kDa) protein expression in liver allograft tissues collected on POD3, POD7 and POD14. GAPDH (37 kDa) served as the internal reference for protein normalization. Numbers beneath each protein band indicate relative expression levels normalized to GAPDH. **(C)** Western blot detection of NR4A1 and FOXP3 protein expression in primary CD4^+^ T cells treated with vehicle Control, TGF-β alone, or TGF-β combined with isoallo-LCA. GAPDH was used for protein normalization, with relative quantification values labeled under each blot band.

*In vitro* tests with isolated primary CD4^+^ T cells demonstrated that TGF-β-mediated upregulation of NR4A1 and FOXP3 was further enhanced when cells were co-cultured with isoallo-LCA (Figure 3C). Joint findings imply that isoallo-LCA may activate NR4A1 signalling to sustain FOXP3 expression and preserve CD4^+^ T cell immune homeostasis.

### Blockade of NR4A1 abrogates isoallo-LCA-conferred liver allograft protection

To verify the necessity of NR4A1 for the protective effects of isoallo-LCA on liver allografts, we performed pharmacological NR4A1 inhibition experiments. Kaplan–Meier survival analysis showed that blocking NR4A1 largely abrogated the survival benefit conferred by isoallo-LCA (log-rank test, *P* = 0.003) (Figure 4A). In line with the survival data, the improved histopathological injury (Figure 4B) and reduced BANFF rejection grades (Figure 4C) seen in isoallo-LCA-treated grafts were markedly reversed following NR4A1 suppression. Moreover, the declines in serum ALT, AST and TBIL triggered by isoallo-LCA were substantially recovered when the NR4A1 inhibitor was co-delivered (Figures 4D–F).

**FIGURE 4:**
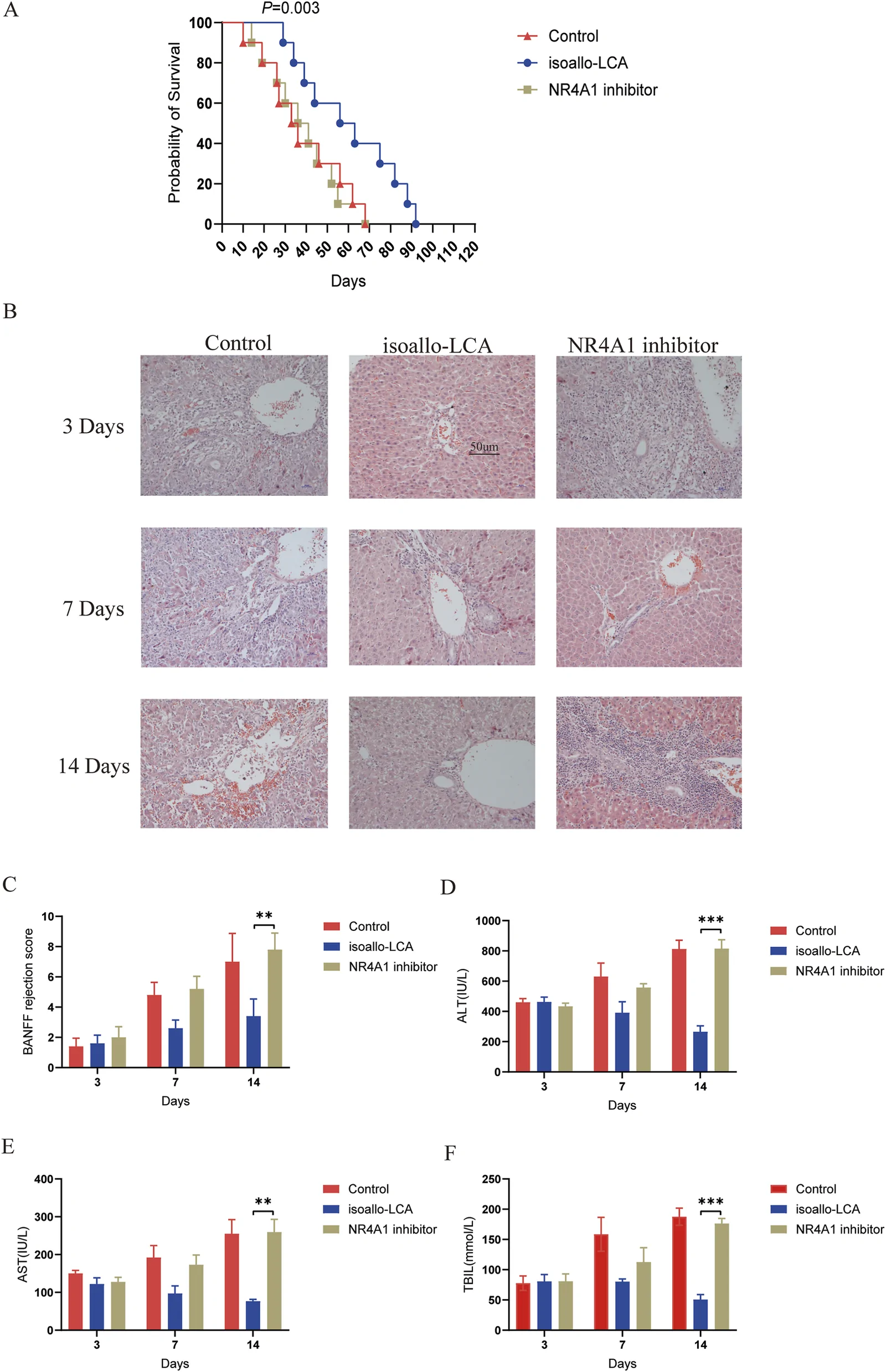
Pharmacological blockade of NR4A1 abolishes the protective tolerance conferred by isoallo-LCA in liver allografts. **(A)** Kaplan–Meier survival curves across three experimental groups: vehicle Control, isoallo-LCA monotherapy, and isoallo-LCA co-administered with NR4A1 inhibitor (n = 10 mice per group). Log-rank test was used for inter-group survival comparison (*P* = 0.003). **(B)** Representative H&E histological micrographs of hepatic graft tissues harvested on POD3, POD7 and POD14 from all three experimental groups. **(C)** Statistical quantification of BANFF rejection scores derived from blinded pathological evaluation (n = 5 biological replicates per group). **(D–F)** Bar charts displaying serum ALT, AST and total bilirubin levels to quantitatively assess hepatocellular injury severity. **P < 0.01, ***P < 0.001.

Together, these pharmacological inhibition assays indicate that NR4A1 is required for isoallo-LCA to mitigate acute liver allograft rejection.

### NR4A1 signaling is essential for isoallo-LCA-driven FOXP3^+^ treg expansion and immune tolerance maintenance

We next explored whether NR4A1 contributes to isoallo-LCA-driven remodelling of intrahepatic Tregs post-transplantation (Figure 5). Flow cytometry showed the percentage of intrahepatic FOXP3^+^CD4^+^ T cells reached 2.30% in the control group. Isoallo-LCA treatment robustly elevated this proportion to 3.87%, whereas co-administration of NR4A1 inhibitor markedly reversed this increase to 2.62% (Figure 5A). Statistical analysis confirmed that the frequency of intrahepatic Tregs was significantly lower in mice receiving both isoallo-LCA and NR4A1 inhibitor relative to the isoallo-LCA monotherapy group (P < 0.01) (Figure 5B).

**FIGURE 5:**
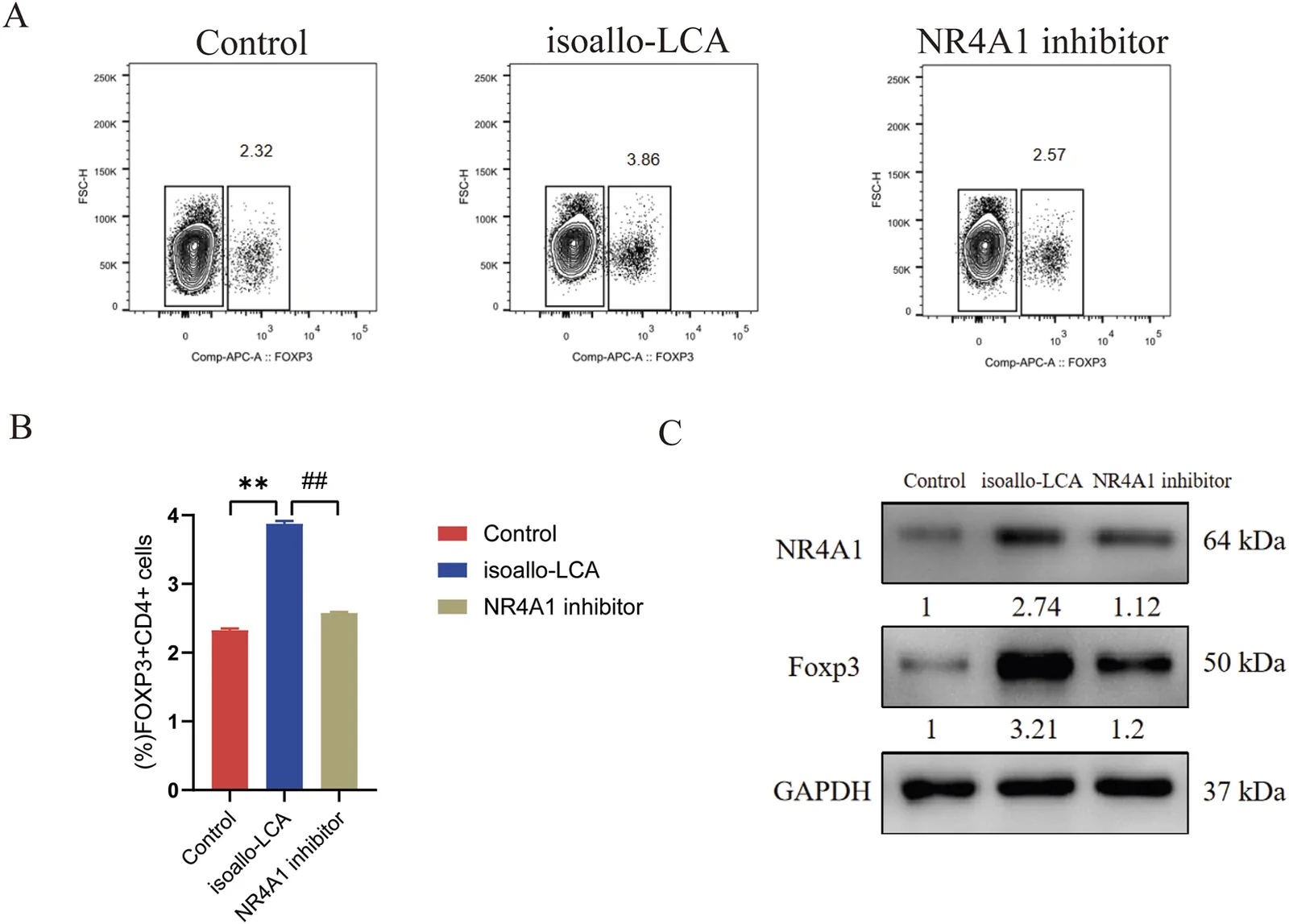
Inhibiting NR4A1 abrogates isoallo-LCA-mediated FOXP3^+^ Treg expansion and suppresses the NR4A1-FOXP3 signaling axis. **(A)** Representative flow cytometry dot plots of FOXP3^+^ CD4^+^ Tregs isolated from liver allografts of vehicle Control, isoallo-LCA monotherapy, and isoallo-LCA + NR4A1 inhibitor groups. Treg percentage values are labeled within each contour plot. **(B)** Bar graph quantifying the frequency of FOXP3^+^ CD4^+^ Tregs across the three experimental cohorts (**P < 0.01). **(C)** Western blot analysis of NR4A1 (64 kDa) and FOXP3 (50 kDa) protein expression in graft-infiltrating lymphocytes isolated from each group. GAPDH (37 kDa) was used as the protein loading control, with normalized relative expression values marked under each protein band.

Western blot analysis of graft-infiltrating lymphocytes further showed that NR4A1 inhibitor treatment downregulated NR4A1 and FOXP3 protein levels, offsetting the upregulatory effect of isoallo-LCA (Figure 5C).

Taken together, these findings indicate that the capacity of isoallo-LCA to expand FOXP3^+^ Tregs and sustain transplant immune tolerance may rely on intact NR4A1 signalling, supporting a functional isoallo-LCA/NR4A1/FOXP3 tolerogenic signaling cascade in orthotopic liver transplantation. It should be noted that transcriptomic enrichment analysis identifies NR4A1 pathway activation as a downstream consequence of isoallo-LCA treatment; whether isoallo-LCA directly interacts with the NR4A1 protein remains to be established through direct binding studies.

## Discussion

This study broadens the biological function of isoallo-LCA to the context of solid organ transplant alloimmunity. Previous studies have primarily characterized the protective effects of isoallo-LCA in intestinal inflammation and autoimmune disorders. Wang et al. (Wang et al., 2025) demonstrated that isoallo-LCA ameliorates intestinal inflammation via metabolically reprogrammed macrophages, while Yue et al. (Yue et al., 2025) showed that isoallo-LCA-modified Treg exosomes alleviate inflammatory bowel disease by inhibiting NF-κB-associated inflammation. However, its immunomodulatory capacity during post-transplant allograft rejection—a fundamentally different immunological context involving alloimmune recognition rather than mucosal inflammation—remains largely uncharacterized (Wang et al., 2025; Yue et al., 2025). Our *in vivo* murine orthotopic liver transplantation data suggest that isoallo-LCA treatment may attenuate hepatic inflammatory injury, preserve liver function, and prolong allograft survival. These findings reveal a potential metabolite-associated protective mechanism against acute liver rejection, extending the immunomodulatory spectrum of isoallo-LCA beyond mucosal immune regulation. It should be noted that intraperitoneal administration was chosen because recipients experience severe intestinal dysfunction and impaired mucosal absorption in the immediate post-transplant period, which would compromise oral bioavailability. Whether this route recapitulates the physiological immunomodulatory effects of gut-derived isoallo-LCA remains an open question for future investigation.

Furthermore, isoallo-LCA exhibits a unique homeostatic immunomodulatory profile that distinguishes it from conventional immunosuppressive agents. Clinical broad-spectrum immunosuppressants alleviate rejection via global T cell suppression, which may compromise systemic immune surveillance and increase the susceptibility to postoperative complications (Lan et al., 2025; Kensiski et al., 2025). In comparison, isoallo-LCA appears to remodel the dysregulated intrahepatic alloimmune microenvironment by modulating the balance between pro-inflammatory Th1 cells and immunosuppressive FOXP3^+^ Tregs, thereby restoring CD4^+^ T cell immune homeostasis without causing generalized immune suppression. This homeostatic regulatory pattern confers improved immune selectivity and biocompatibility to isoallo-LCA, highlighting its potential as a refined immunomodulatory strategy for sustaining post-transplant immune tolerance.

Mechanistically, the present study identifies and validates the isoallo-LCA/NR4A1/FOXP3 tolerogenic signaling axis governing liver transplant immunity based on murine experimental systems. Integrative transcriptomic profiling, *in vivo* murine orthotopic liver transplantation models, *in vitro* cellular functional assays, and pharmacological intervention experiments demonstrate that intact NR4A1 signaling is indispensable for isoallo-LCA to upregulate FOXP3 expression and facilitate Treg expansion. Pharmacological inhibition of NR4A1 substantially diminishes the protective effects of isoallo-LCA against allograft rejection in mice, demonstrating that NR4A1 is an essential and indispensable mediator of isoallo-LCA’s protective effects. The near-complete reversal of survival benefit, histological improvement, and Treg expansion upon NR4A1 blockade provides the strongest evidence for a causal role of this metabolite-dependent signaling cascade in regulating transplant tolerance, rather than a mere correlative association. These observations partially fill existing research gaps regarding metabolite-mediated immune regulation in liver transplantation and provide a promising candidate target for future translational anti-rejection research. It should be noted that while the absolute increase in Treg proportion (approximately 1.5 percentage points) may appear modest, even small shifts in the Th1/Treg balance can have substantial functional consequences in the transplant microenvironment. Moreover, the concurrent reduction in pro-inflammatory Th1 cells from 14.57% to 6.38% represents a more substantial shift that, combined with Treg expansion, produces a meaningful net shift toward immune tolerance.

Collectively, this work presents multi-layered findings regarding the previously unrecognized protective effect of isoallo-LCA against acute liver allograft rejection, its unique homeostatic immune regulatory characteristics, and the involvement of the NR4A1/FOXP3 signaling axis. As an endogenous, low-toxic gut microbial metabolite, isoallo-LCA holds promising potential as an adjuvant therapeutic agent for post-transplant rejection management. Nevertheless, several limitations should be acknowledged. This study mainly focuses on CD4^+^ T cell subsets, while the regulatory effects of isoallo-LCA on intrahepatic macrophages, dendritic cells, and CD8^+^ T cells remain to be clarified. The precise molecular mechanism by which isoallo-LCA modulates NR4A1 activation also requires further investigation. Additionally, all experiments were conducted in murine models, and whether these findings can be extrapolated to human liver transplantation needs further clinical validation. This study only explores the therapeutic effect of isoallo-LCA on acute rejection, and its long-term impact on chronic allograft injury remains unclear. Furthermore, while the present study focuses on the NR4A1/FOXP3 axis, Treg differentiation and maintenance are regulated by multiple parallel signaling pathways, including IL-2/STAT5, TGF-β/Smad, and mTOR signaling; whether isoallo-LCA acts solely through NR4A1 or also engages these additional pathways remains to be determined. Cross-comparison with other classic bile acids is also warranted: while UDCA primarily exerts cytoprotective effects and LCA/DCA act as FXR/TGR5 agonists, isoallo-LCA uniquely promotes NR4A1-mediated Treg differentiation, representing a distinct tolerogenic mechanism. Notably, the enrichment of isoallo-LCA-producing gut microbiota in centenarians (Sato et al., 2021) suggests a natural tolerance-maintaining mechanism that could be therapeutically harnessed.

### Translational prospects and clinical feasibility

The present findings raise several important translational considerations. First, gut microbiome composition varies substantially among individuals, potentially leading to different endogenous isoallo-LCA levels that could influence rejection risk. Second, the homeostatic immunomodulatory profile of isoallo-LCA—selective Th1 suppression and Treg promotion without generalized immune suppression—positions it as an ideal adjunct agent to reduce the dosage and side effects of conventional immunosuppressants. Third, future clinical trials could evaluate isoallo-LCA as add-on therapy in the early post-transplant period. In summary, this study indicates that isoallo-LCA may mitigate acute liver allograft rejection and improve graft outcomes by activating the NR4A1/FOXP3 axis to restore intrahepatic CD4^+^ T cell immune homeostasis. Our results provide additional *in vivo* evidence for the immunomodulatory potential of gut microbial bile acid metabolites in liver transplant settings, offer novel mechanistic insights into alloimmune tolerance regulation, and may inform the development of future targeted anti-rejection strategies.

## Data Availability

The original contributions presented in the study are publicly available. This data can be found here: https://www.ncbi.nlm.nih.gov/bioproject/PRJNA1522992.

## References

[B1] EASL clinical practice guidelines on liver transplantation. (2024). J. Hepatol. 81 (6), 1040–1086. 10.1016/j.jhep.2024.07.032 39487043

[B2] Kensiski A. Gavzy S. J. Wu L. Mas V. Ma B. Bromberg J. S. (2025). Immunosuppressant imprecision: multidirectional effects on metabolism and microbiome. Clin. Microbiol. Rev. 38 (2), e0017824. 10.1128/cmr.00178-24 40042298 PMC12160495

[B3] Lan T. Wu G. Zuo B. Yang J. He P. Zhang Y. (2025). Regulation of the immune microenvironment and immunotherapy after liver transplantation. Front. Immunol. 16, 1602877. 10.3389/fimmu.2025.1602877 40421010 PMC12104065

[B4] Li L. Wang J. Huang L. Chen Y. Chen L. (2025). Inhibition of HMGB1/NF-κB signaling restores Th17/Treg balance via dendritic cell modulation in liver transplant rejection. Front. Immunol. 16, 1649366. 10.3389/fimmu.2025.1649366 40977677 PMC12444659

[B5] Li Q. X. Zhang J. X. Li H. Li X. L. He Q. Han D. D. (2026). A pre-emptive risk model for acute rejection in liver transplantation: an immunopharmacologic biomarker panel combining CD4+ T-cell profiling and tacrolimus exposure. Front. Immunol. 17, 1760409. 10.3389/fimmu.2026.1760409 41853285 PMC12992057

[B6] Mehtani R. Saigal S. (2023). Long term complications of immunosuppression post liver transplant. J. Clin. Exp. Hepatol. 13 (6), 1103–1115. 10.1016/j.jceh.2023.06.007 37975039 PMC10643541

[B7] Rabiee A. Girone G. Davis J. P. E. (2023). Brief overview of immunosuppression and its side effects after liver transplantation. Clin. Liver Dis. (Hoboken) 21 (6), 160–164. 10.1097/CLD.0000000000000035 37937047 PMC10627593

[B8] Ronca V. Wootton G. Milani C. Cain O. (2020). The immunological basis of liver allograft rejection. Front. Immunol. 11, 2155. 10.3389/fimmu.2020.02155 32983177 PMC7492390

[B9] Sato Y. Atarashi K. Plichta D. R. Arai Y. Sasajima S. Kearney S. M. (2021). Novel bile acid biosynthetic pathways are enriched in the microbiome of centenarians. Nature 599 (7885), 458–464. 10.1038/s41586-021-03832-5 34325466

[B10] Wang Y. Yan W. Zhao H. Lu Y. Li Y. Peng S. (2025). Isoallolithocholic acid ameliorates intestinal inflammation via metabolically reprogrammed macrophages. Commun. Biol. 8 (1), 1718. 10.1038/s42003-025-09123-3 41315591 PMC12663217

[B11] Yokota S. Ueki S. Ono Y. Kasahara N. Pérez-Gutiérrez A. Kimura S. (2016). Orthotopic mouse liver transplantation to study liver biology and allograft tolerance. Nat. Protoc. 11 (7), 1163–1174. 10.1038/nprot.2016.073 27254462

[B12] Yokota S. Li W. Clavien P. A. Kupiec-Weglinski J. W. Thomson A. W. (2025). Mouse orthotopic liver transplantation: challenges, achievements, and applications. Am. J. Transpl. 25 (9), 1830–1842. 10.1016/j.ajt.2025.06.011 40523479 PMC13222681

[B13] Yue S. Gong L. Tan Y. Zhang X. Liao F. (2025). IsoalloLCA-intervened regulatory T cell exosomes alleviate inflammatory bowel disease by inhibiting NF-κB-associated inflammation in intestinal epithelial cells. Int. Immunopharmacol. 154, 114501. 10.1016/j.intimp.2025.114501 40174336

[B14] Zhao K. Wang R. Kamoun M. Callans L. Bremner R. Rame E. (2022). Incidence of acute rejection and patient survival in combined heart-liver transplantation. Liver Transpl. 28 (9), 1500–1508. 10.1002/lt.26448 35247292 PMC12063157

[B15] Zhou A. W. Jin J. Liu Y. (2024). Cellular strategies to induce immune tolerance after liver transplantation: clinical perspectives. World J. Gastroenterol. 30 (13), 1791–1800. 10.3748/wjg.v30.i13.1791 38659486 PMC11036497

[B16] Zhou X. Li X. Jiang P. Li S. Jia Z. Wang X. (2026). LDLR(+) monocytic myeloid-derived suppressor cells attenuate allograft rejection in liver transplantation. J. Clin. Transl. Hepatol. 14 (3), 301–316. 10.14218/JCTH.2025.00621 41970191 PMC13063287

